# Association between dietary selenium intake and the prevalence of prediabetes in Newfoundland population: a cross-sectional study

**DOI:** 10.3389/fnut.2025.1615462

**Published:** 2025-08-26

**Authors:** Shanshan Yu, Hongwei Zhang, Jianling Du, Guang Sun

**Affiliations:** ^1^Department of Endocrinology, First Affiliated Hospital of Dalian Medical University, Dalian, China; ^2^Discipline of Medicine, Faculty of Medicine, Memorial University of Newfoundland, St. John’s, NL, Canada

**Keywords:** selenium, prediabetes, obesity, threshold effect, CODING study

## Abstract

**Introduction:**

Emerging evidence highlights the role of selenium (Se) in glucose metabolism through selenoprotein-mediated antioxidant and anti-inflammatory pathways. However, population-specific data remains inconclusive. This study aims to investigate the association between dietary Se intake and prediabetes prevalence in Newfoundland, a population characterized by genetic homogeneity and high obesity rates (39.4%).

**Methods:**

This cross-sectional study used data from 2,665 participants in the Complex Diseases in the Newfoundland Population: Environment and Genetics (CODING) study. Prediabetes was defined by the American Diabetes Association criteria for impaired fasting glucose (FPG: 5.6–6.9 mmol/L). Dietary Se intake was assessed using the Willett food frequency questionnaire and expressed as both absolute (μg/d) and body weight-adjusted (μg/kg/d) metrics. Multivariate logistic regression, generalized additive model regression, piecewise regression models, and subgroup stratification were employed to examine the association.

**Results:**

The study revealed a significant inverse relationship between body weight-adjusted dietary Se intake (μg/kg/d) and prediabetes prevalence in the fully adjusted models, with a non-linear threshold effect observed at 1.42 μg/kg/d. Below this threshold, each 1-unit increase in dietary Se intake (μg/kg/d) reduced prediabetes risk by 69% (OR = 0.31, *P* < 0.001). However, such an association did not reach statistical significance beyond 1.42 μg/kg/d. Subgroup analyses demonstrated consistent inverse associations across age groups, family history of diabetes, and history of smoking. However, the association was statistically significant in females (OR = 0.10, *p* < 0.001) but not in males. Absolute dietary Se intake (μg/d) showed no significant correlation with prediabetes after adjustment.

**Discussion:**

Weight-adjusted dietary Se intake (μg/kg/d) exhibits an inverse non-linear, threshold-dependent relationship with prediabetes risk in this high-risk population. The findings underscore the critical importance of body weight normalization in assessing Se’s metabolic effects and formulating Se guidelines.

## Introduction

Prediabetes, a precursor to type 2 diabetes mellitus (T2DM), is defined by the American Diabetes Association (ADA) as impaired fasting glucose (IFG: 5.6 ~ 6.9 mmol/L), glycated hemoglobin level (HbA_1C_) of 5.7 ~ 6.4%, or impaired glucose tolerance (IGT: 2-h postprandial glucose 7.8 ~ 11.0 mmol/L) (1). This metabolic intermediate state affects over 14.9% (762 million) adults globally in 2021, with projections estimating a rise to 16.5% (1,052 million) by 2045 (2). Moreover, this age-adjusted prevalence of prediabetes was highest in the North America and Caribbean region, at 20.7% (75 million) in 2021 (2). Notably, people with prediabetes are at elevated risk for developing diabetes, with up to 50% progressing to diabetes within 5 years (2, 3). People with prediabetes are also at increased risk of all-cause mortality, cardiovascular disease, and microvascular complications (2, 4). Thus, identifying modifiable factors linked to prediabetes and intervening early is critical to minimize the long-term medical burden of pre-diabetes.

Emerging evidence highlights dietary micronutrients, particularly selenium (Se), as modifiable determinants of glucose metabolism. Se exerts its biological function mainly through selenoproteins, which are critical for mitigating oxidative stress and inflammation, processes that are implicated in *β*-cell dysfunction and insulin resistance (5, 6). However, the relationship between dietary Se intake and diabetes remains inconclusive. A prospective study from Northern Italy showed that increased dietary selenium intake was associated with an increased risk of T2DM (7), while a Brazilian study found no association between Se intake and diabetes (8). Moreover, a study using data from the China Health and Nutrition Survey (CHNS) recently reported a V-shaped relationship where both low and high dietary Se intakes correlated with elevated diabetes risk, with the lowest risk observed at 45 μg/d (9). In addition, a meta-analysis of 20 randomized controlled trials has indicated the potential beneficial effects of selenium supplementation on fasting insulin levels and insulin sensitivity (10). These discrepancies underscore the need for population-specific analyses, particularly in regions with unique genetic or environmental predispositions.

The Newfoundland population presents unique genetic and environmental characteristics that enhance its suitability for investigating Se’s metabolic role. Genetically, the population exhibits reduced diversity due to founder effects and prolonged geographic isolation, resulting in a homogeneous genetic background (11, 12). Environmentally, Newfoundland has the highest obesity rates in Canada (39.4% vs. the national average of 27.2%) and elevated insulin resistance, driven by unique dietary patterns and low physical activity levels (13). Our previous analyses from the Complex Diseases in the Newfoundland Population: Environment and Genetics (CODING) study revealed significant inverse associations between dietary Se intake and body fat measured by dual-energy X-ray absorptiometry (DXA), as well as insulin resistance determined by the homeostasis model assessment (HOMA-IR) (14, 15). These findings highlight Se’s metabolic relevance in this cohort. However, the role of dietary Se intake in prediabetes in this high-risk group has not yet been reported.

This cross-sectional study investigates the association between dietary Se intake and prediabetes prevalence in a high-risk Newfoundland population. Dietary Se intake is evaluated using both absolute (μg/d) and body weight-adjusted (μg/kg/d) metrics in this study. By employing standardized ADA diagnostic criteria for IFG and adjusting for key confounders, our findings aim to clarify the role of Se intake in early dysglycemia and to identify population-specific dietary recommendations for at-risk populations.

## Methods

### Study population

This study was conducted using data from the Complex Diseases in the Newfoundland population: Environment and Genetics (CODING) study database. Eligibility of participants for the CODING study was based upon the following inclusion criteria: (1) ≥ 19 years of age; (2) at least a third generation Newfoundlander; (3) healthy, without any serious metabolic, cardiovascular, or endocrine diseases; and (4) women were not pregnant at the time of the study (14–16). The CODING study was approved by the Health Research Ethics Authority (HREA) of Newfoundland, St. John’s, Canada, with project identification code #10.33. All subjects provided written informed consent and all methods were performed in accordance with the relevant guidelines and regulations.

**Figure 1:**
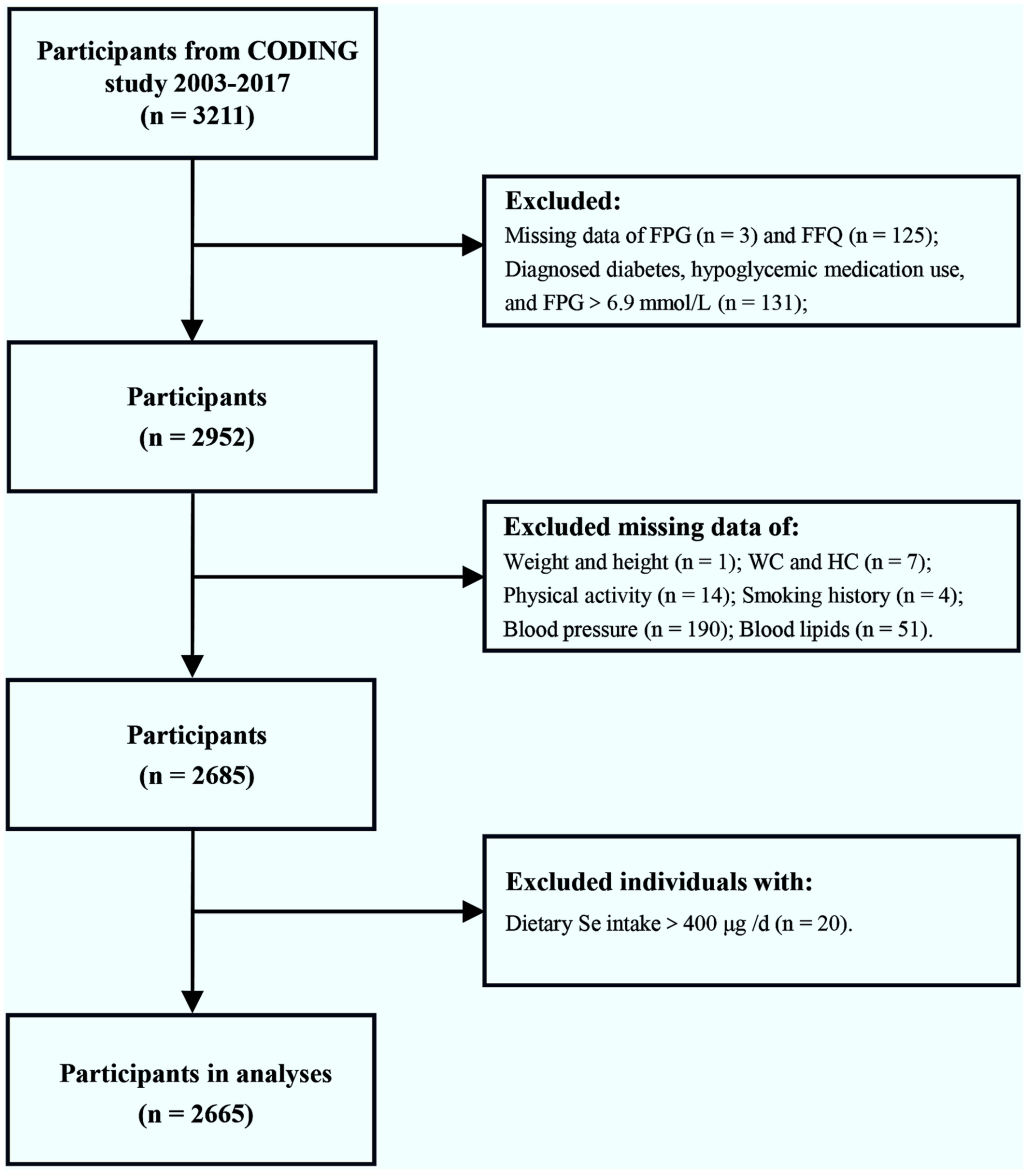
Flow chart of the enrolled participants. Initially, a total of 3,211 participants from the CODING study (2003–2017) were included. After exclusion of individuals with diagnosed diabetes, hypoglycemic medication use, FPG>6.9 mmol/L, dietary Se intake >400 μg/d, or incomplete/missing data on FPG, FFQ, and relevant covariates, a final total of 2,665 participants were included in the analysis. FPG, fasting plasma glucose; FFQ, food frequency questionnaire; WC, waist circumference; HC, hip circumference; Se, selenium.

At baseline, a total of 3,211 participants from the CODING study, conducted between 2003 and 2017, were included. Figure 1 depicts the procedure of participants’ selection. We excluded individuals who did not have data on fasting plasma glucose (FPG) (*n* = 3), those who self-reported diagnosed diabetes, those who used any hypoglycemic medications, and those with FPG>6.9 mmol/L (*n* = 131). Furthermore, 125 individuals lacking data on the food frequency questionnaire (FFQ) were excluded. We further excluded individuals with missing covariate data (*n* = 267), as follows: 1 individual missing data on weight and height, 7 individuals missing data on waist circumference (WC) and hip circumference (HC), 14 individuals missing physical activity information, 4 individuals missing smoking history information, 190 individuals missing blood pressure data, and 51 individuals missing blood lipids data. In addition, 20 individuals with dietary Se intake above 400 μg per day, the upper tolerable limit for adults (17, 18), were excluded. In the end, 2,665 participants were included in our analysis. To address potential selection bias, we compared participants excluded solely due to missing covariate data (*n* = 267) to the final analytical sample (*n* = 2,665) on key characteristics that were available for both groups prior to the point of exclusion: age, sex, FPG, caloric intake, and dietary Se intake. We found no statistically significant differences between the excluded group and the final analytical sample for any of these available characteristics (*p* > 0.05 for all comparisons; detailed data provided in Supplementary Table 1). Comparisons on specific covariates that were missing (data on weight, WC, HC, physical activity, smoking history, blood pressure, and blood lipids) were not possible, as these data were absent for the excluded group. While we cannot rule out differences on these specific missing covariates, the lack of significant differences on the available key demographic, metabolic (FPG), and dietary factors offers reassurance that the exclusion did not introduce substantial bias on these available fundamental characteristics. These findings support the representativeness of the final sample regarding these available factors.

### Dietary se intake

Dietary intake for each participant was assessed using a 124-item semiquantitative Willett FFQ, which is the most cost-effective method commonly used in large-scale epidemiological studies (19, 20). Although the original FFQ design prioritized nutrients associated with cancer and cardiovascular disease (19), we ensured comprehensive assessment of Se by including regionally relevant Se-rich foods commonly consumed in the Newfoundland population, such as cod and shellfish, based on Canadian Nutrient File (CNF). Participants reported weekly consumption frequencies for food items over the preceding 12 months. Daily intake values were derived by converting portion sizes to gram equivalents. Absolute Se intake (μg/d) and caloric intake (kcal/d) were calculated using Nutribase Clinical Nutrition Manager (software version 9.0; Cybersoft Inc., Phoenix, AZ, United States) and its integrated US Department of Agriculture (USDA) Standard Reference (SR) database and CNF. We verified that the USDA SR and CNF include the selenium content of key local foods, ensuring accurate representation of the population’s dietary patterns (Comments d). We used absolute intake metrics (μg/d) without energy adjustment based on the following considerations:

1. Biological relevance: The biological effects of Se depend on absolute intake levels due to the saturable nature of selenoprotein synthesis and the existence of a threshold for optimal status (e.g., glutathione peroxidase, selenoprotein P) (21).
2. Precedent in Se research: major studies on Se and health outcomes, including the Nutritional Prevention of Cancer (NPC) trial and the Se and Vitamin E Cancer Prevention Trial (SELECT), as well as clinical guidelines prioritize absolute intake (μg/d) as the primary metric for Se status assessment (17, 18, 22–25).
3. Validation in our previous work: Our research group has consistently demonstrated significant associations between absolute dietary Se intake (μg/d) and health outcomes (e.g., reduced body fat and improved insulin resistance) in the CODING study using the same methodology (14, 15).

Additionally, Se intake was expressed as μg/kg/d to account for body size variability, consistent with methodological approaches in our prior Se trials (14, 15).

### Diagnosis of prediabetes

In this study, the diagnostic criteria of IFG-based prediabetes was according to the standards recommended by ADA Professional Practice Committee in 2025, with the definition of FPG levels ranging from 5.6 to 6.9 mmol/L (1).

### Anthropometric data and other information

All anthropometric data were measured and recorded by trained personnel from all participants following a 12 h overnight fast, including sex, age, body weight, height, waist circumference (WC), and hip circumference (HC). Body mass index (BMI) = body weight /height^2^ (kg/m^2^). Waist-to-hip ratio (WHR) = WC/ HC. Measurements of systolic blood pressure (SBP) and diastolic blood pressure (DBP) were also acquired by trained personnel. Moreover, physical activity levels were measured by ARIC Baecke Questionnaire, which consists of work, sport, and leisure time activity indices (14, 15). Additionally, all participants completed a self-administered screening questionnaire for collecting information about their personal and their parents’ health history.

### Measurements of laboratory data

Blood samples were collected from all participants following a 12 h overnight fasting. Plasma and serum samples were isolated from whole blood for subsequent analyses. Concentrations of fasting plasma glucose (FPG), triacylglycerols (TG), total cholesterol (TC), and high-density lipoprotein cholesterol (HDL-C) were measured with the use of Synchron reagents and performed on an Lx20 analyzer (Beckman Coulter, Brea, CA). Low-density lipoprotein cholesterol (LDL-C) was calculated using the following formula: [TC - HDL-C - (TG/2.2)] which is reliable in the absence of severe hyperlipidemia. The triglyceride glucose (TyG) index was calculated as Ln [TG (mg/dl) × FPG (mg/dl)/2].

### Statistical analyses

SPSS software version 26.0 and EmpowerStats software^1^ were used for statistical analyses. The normality of all continuous variables was assessed using the Shapiro–Wilk test. Continuous variables that were not normally distributed (caloric intake, TG, and dietary Se intake) were log-transformed to normalize distributions for subsequent statistical analyses. However, for descriptive purposes, all continuous variables are presented as mean ± standard error (SE) in their original units. Categorical variables were presented as frequency counts and percentages. Comparison of continuous data between two subgroups were analyzed using the independent Student’s t-test. Comparisons of continuous data among quartiles of dietary Se intake were performed using one-way analysis of variance (ANOVA) test, followed by pairwise comparisons using least significant difference (LSD). The χ^2^ test was used for comparison of percentages among groups for categorical data.

The associations between dietary Se intake, various dietary Se intake quartiles, and the prevalence of prediabetes were examined using multivariate logistic regression models. Dietary Se intake, when used as a continuous variable, underwent log 10 transformation in the logistic regression analyses. Model 1 was the crude model without adjustment for potential confounders. Model 2 was adjusted for age and sex. Model 3 was further adjusted for physical activity, caloric intake, family history of diabetes (categorized as yes, no, or not recorded), and history of smoking. For the covariate family history of diabetes (which included 161 participants with “Not recorded” data), a separate category (“not recorded”) was created and retained in all regression models to preserve sample size and avoid exclusion of cases. Additionally, the non-linear dose–response relationship between dietary Se intake (μg/kg/d) and the prevalence of prediabetes was assessed through generalized additive model regression and smooth curve fitting methods, and the threshold effect analysis was addressed by two-piecewise linear regression. Subgroup analyses were further performed based on the stratified factors including sex, age, family history of diabetes, and history of smoking. Statistical significance was considered at *p* < 0.05.

## Results

### Basic characteristics of the study population according to pre-diabetes status

A total of 2,665 participants were included in the present study, among whom 365 were identified as participants with prediabetes (Table 1). The average dietary Se intake of the entire population was 106.20 μg/d, with its level 107.32 μg/d in non-prediabetes subjects and 99.15 μg/d in prediabetes subjects. Similarly, this significant difference persisted after dietary Se intake adjusted for body weight, with its level lower in subjects with prediabetes than non-prediabetes (1.27 μg/kg/d vs. 1.52 μg/kg/d, *P*<0.001). Notable distinctions were also observed between the non-prediabetes and prediabetes groups, including age, sex, body weight, BMI, WC, WHR, physical activity, caloric intake, and family history of diabetes. In addition, subjects with prediabetes were more likely to have higher SBP, DBP, FPG, TG, TC, LDL-C, and lower HDL-C, as well as a higher TyG index, which has been recognized as a valuable surrogate marker of insulin resistance.

**Table 1:** Basic characteristics of the study population according to prediabetes status.

Variables	Entire cohort (*n* = 2,665)	Non-prediabetes (*n* = 2,300)	Prediabetes (*n* = 365)	*p* value
Age (years)	43.28 ± 0.24	42.16 ± 0.26	50.38 ± 0.57	<0.001
Sex, *n* (%)				<0.001
Male	714 (26.79%)	574 (24.96%)	140 (38.36%)	
Female	1951 (73.21%)	1726 (75.04%)	225 (61.64%)	
Weight (kg)	73.86 ± 0.31	72.73 ± 0.32	81.01 ± 0.91	<0.001
BMI (kg/m^2^)	26.58 ± 0.09	26.20 ± 0.10	28.98 ± 0.28	<0.001
WC (cm)	91.77 ± 0.27	90.54 ± 0.28	99.48 ± 0.74	<0.001
WHR	0.92 ± 0.001	0.91 ± 0.002	0.94 ± 0.004	<0.001
Physical activity	8.28 ± 0.03	8.34 ± 0.03	7.88 ± 0.08	<0.001
Caloric intake (kcal/d)^a^	1952.49 ± 16.32	1968.31 ± 17.83	1852.78 ± 39.32	0.017
SBP (mmHg)	123.43 ± 0.31	122.19 ± 0.32	131.26 ± 0.88	<0.001
DBP (mmHg)	80.63 ± 0.21	80.00 ± 0.22	84.59 ± 0.60	<0.001
FPG (mmol/L)	5.02 ± 0.01	4.89 ± 0.01	5.90 ± 0.02	<0.001
TG (mmol/L)^a^	1.17 ± 0.01	1.11 ± 0.01	1.54 ± 0.05	<0.001
TC (mmol/L)	5.12 ± 0.02	5.05 ± 0.02	5.52 ± 0.06	<0.001
HDL-C (mmol/L)	1.46 ± 0.01	1.48 ± 0.01	1.40 ± 0.02	<0.001
LDL-C (mmol/L)	3.10 ± 0.02	3.05 ± 0.02	3.42 ± 0.05	<0.001
TyG index	8.29 ± 0.01	8.22 ± 0.01	8.73 ± 0.03	<0.001
Family history of diabetes, *n* (%)				0.021
Yes	505 (18.95%)	417 (18.13%)	88 (24.11%)	
No	1999 (75.01%)	1740 (75.65%)	259 (70.96%)	
Not recorded	161 (6.04%)	143 (6.22%)	18 (4.93%)	
History of smoking, *n* (%)				0.84
Yes	291 (10.92%)	250 (10.87%)	41 (11.23%)	
No	2,374 (89.08%)	2050 (89.13%)	324 (88.77%)	
Dietary Se intake				
Se intake (μg/d)^a^	106.20 ± 1.00	107.32 ± 1.08	99.15 ± 2.54	0.001
Se intake (μg/kg/d)^a^	1.49 ± 0.01	1.52 ± 0.02	1.27 ± 0.03	<0.001

^a^Caloric intake, TG, and dietary Se intake were log-transformed to normalize distributions for subsequent independent Student’s t-test, however, for descriptive purposes, they were presented as mean ± SE in their original units. BMI, body mass index; WC, waist circumference; WHR, waist-to-hip ratio; SBP, systolic blood pressure; DBP, diastolic blood pressure; FPG, fasting plasma glucose; TG, triacylglycerols; TC, total cholesterol; HDL-C, high-density lipoprotein cholesterol; LDL-C, low-density lipoprotein cholesterol; TyG index, triglyceride-glucose index; Se, selenium; SE, standard error.

### Clinical characteristics of subjects according to dietary se quartiles

The comparisons of clinical characteristics were performed among quartiles of dietary Se intake, expressed as micrograms per kilogram per day (μg/kg/d) and micrograms per day (μg/d), respectively (Tables 2, 3). This study demonstrated that the prevalence of prediabetes decreased with increasing quartiles of dietary Se intake expressed as μg/kg/d, with its level 19.85, 15.3, 10.04, and 9.61% in quartiles 1, 2, 3, and 4, respectively (*p*<0.001; Table 2). It also showed that participants with higher dietary Se intake were with decreased body weight, BMI, WC, WHR, SBP, DBP, FPG, TG, TC, LDL-C, TyG index, and increased HDL-C (*p*<0.05 for all).

**Table 2:** Clinical characteristics according to quartiles of dietary Se intake (μg/kg/d).

Variables	Dietary Se intake (μg/kg/d)				*p* value
	Q1	Q2	Q3	Q4	
Number (*n* = 2,665)	665	667	667	666	
Se (μg/kg/d)	0.16 ~ 0.98	0.99 ~ 1.34	1.35 ~ 1.82	1.83 ~ 6.69	
Prediabetes, *n* (%)	132 (19.85%)	102 (15.3%)	67 (10.04%)	64 (9.61%)	<0.001
Weight (kg)	81.06 ± 0.70	76.19 ± 0.63^**a**^	71.40 ± 0.53^**ab**^	66.81 ± 0.47^**abc**^	<0.001
BMI (kg/m^2^)	29.00 ± 0.21	27.30 ± 0.18^**a**^	25.80 ± 0.16^**ab**^	24.23 ± 0.14^**abc**^	<0.001
WC (cm)	99.10 ± 0.58	93.88 ± 0.52^**a**^	89.24 ± 0.45^**ab**^	84.86 ± 0.42^**abc**^	<0.001
WHR	0.93 ± 0.003	0.92 ± 0.003^**a**^	0.90 ± 0.003^**ab**^	0.90 ± 0.003^**ab**^	<0.001
SBP (mmHg)	126.93 ± 0.66	123.79 ± 0.63^**a**^	121.76 ± 0.59^**ab**^	121.26 ± 0.57^**ab**^	<0.001
DBP (mmHg)	83.22 ± 0.46	80.80 ± 0.41^**a**^	79.67 ± 0.39^**a**^	78.83 ± 0.42^**ab**^	<0.001
FPG (mmol/L)	5.13 ± 0.02	5.05 ± 0.02^**a**^	4.96 ± 0.02^**ab**^	4.93 ± 0.02^**ab**^	<0.001
TG (mmol/L)	1.37 ± 0.03	1.22 ± 0.03^**a**^	1.04 ± 0.02^**ab**^	1.02 ± 0.03^**ab**^	<0.001
TC (mmol/L)	5.21 ± 0.04	5.19 ± 0.04^**a**^	5.05 ± 0.04^**ab**^	5.02 ± 0.04^**ab**^	0.001
HDL-C (mmol/L)	1.39 ± 0.01	1.44 ± 0.01^**a**^	1.51 ± 0.02^**ab**^	1.52 ± 0.01^**ab**^	<0.001
LDL-C (mmol/L)	3.16 ± 0.04	3.16 ± 0.03	3.04 ± 0.03^**ab**^	3.02 ± 0.03^**ab**^	0.002
TyG index	8.49 ± 0.02	8.36 ± 0.02^**a**^	8.19 ± 0.02^**ab**^	8.15 ± 0.02^**ab**^	<0.001

^a^Compared with Q1, *p* < 0.05; ^b^compared with Q2, *p* < 0.05; ^c^compared with Q3, *p* < 0.05. Se, selenium; BMI, body mass index; WC, waist circumference; WHR, waist-to-hip ratio; SBP, systolic blood pressure; DBP, diastolic blood pressure; FPG, fasting plasma glucose; TG, triacylglycerols; TC, total cholesterol; HDL-C, high-density lipoprotein cholesterol; LDL-C, low-density lipoprotein cholesterol; TyG index, triglyceride-glucose index.

**Table 3:** Clinical characteristics according to quartiles of dietary Se intake (μg/d).

Variables	Dietary Se intake (μg/d)				*p* value
	Q1	Q2	Q3	Q4	
Number (*n* = 2,665)	666	667	666	666	
Se (μg/d)	10.60 ~ 72.36	72.37 ~ 97.54	97.55 ~ 128.28	128.29 ~ 399.63	
Prediabetes, *n* (%)	115 (17.27%)	95 (14.24%)	72 (10.81%)	83 (12.46%)	0.005
Weight (kg)	72.10 ± 0.60	72.78 ± 0.59	74.97 ± 0.64^**ab**^	75.60 ± 0.65^**ab**^	<0.001
BMI (kg/m^2^)	26.60 ± 0.19	26.57 ± 0.18	26.70 ± 0.20	26.46 ± 0.19	0.85
WC (cm)	92.36 ± 0.53	91.91 ± 0.54	91.89 ± 0.55	90.91 ± 0.53	0.276
WHR	0.91 ± 0.003	0.91 ± 0.003	0.91 ± 0.003	0.92 ± 0.003^**abc**^	0.007
SBP (mmHg)	123.58 ± 0.66	122.90 ± 0.64	123.39 ± 0.60	123.85 ± 0.57	0.742
DBP (mmHg)	81.30 ± 0.44	80.63 ± 0.42	80.41 ± 0.41	80.18 ± 0.43	0.279
FPG (mmol/L)	5.05 ± 0.02	5.01 ± 0.02	4.99 ± 0.02	5.02 ± 0.02	0.213
TG (mmol/L)	1.28 ± 0.03	1.14 ± 0.03^**a**^	1.13 ± 0.03^**a**^	1.11 ± 0.03^**a**^	<0.001
TC (mmol/L)	5.23 ± 0.04	5.13 ± 0.04	5.06 ± 0.04^**a**^	5.04 ± 0.04^**a**^	0.002
HDL-C (mmol/L)	1.48 ± 0.02	1.50 ± 0.02	1.45 ± 0.02	1.43 ± 0.01^**ab**^	0.003
LDL-C (mmol/L)	3.15 ± 0.04	3.07 ± 0.03	3.08 ± 0.03	3.09 ± 0.03	0.297
TyG index	8.38 ± 0.02	8.27 ± 0.02^**a**^	8.26 ± 0.02^**a**^	8.24 ± 0.02^**a**^	<0.001

^a^Compared with Q1, *p* < 0.05; ^b^compared with Q2, *p* < 0.05; ^c^compared with Q3, *p* < 0.05. Se, selenium; BMI, body mass index; WC, waist circumference; WHR, waist-to-hip ratio; SBP, systolic blood pressure; DBP, diastolic blood pressure; FPG, fasting plasma glucose; TG, triacylglycerols; TC, total cholesterol; HDL-C, high-density lipoprotein cholesterol; LDL-C, low-density lipoprotein cholesterol; TyG index, triglyceride-glucose index.

Similar results were shown for the prevalence of prediabetes when dietary Se intake was expressed as μg/d (Table 3). However, slightly increased body weight and WHR, as well as decreased HDL-C, were shown with higher dietary Se intake. The differences in BMI, WC, SBP, DBP, FPG, and LDL-C were not statistically significant among the four dietary Se intake groups.

### Logistic regression analysis between dietary se intake and the prevalence of prediabetes

The multivariate logistic regression analysis of correlations between the prevalence of prediabetes and dietary Se intake, expressed as μg/kg/d and μg/d, were presented, respectively (Table 4). When dietary Se intake was expressed as μg/kg/d, it showed that log-transformed dietary Se intake was inversely correlated with the prevalence of prediabetes. This negative relationship persisted in the fully adjusted model (Model 3, OR = 0.15, 95% CI: 0.07 ~ 0.36, *p*<0.001), indicating that for every unit increase in log-transformed dietary Se intake (μg/kg/d), the prevalence of prediabetes reduces by 85%. In addition, sensitivity analyses using dietary Se intake (μg/kg/d) as a categorical variable (quartiles) also supported the above findings. Subjects in Q3 (1.35 ~ 1.82 μg/kg/d) and Q4 (1.83 ~ 6.69 μg/kg/d) showed 48% (OR = 0.52, 95% CI: 0.36 ~ 0.77, *p* = 0.001) and 46% (OR = 0.54, 95% CI: 0.35 ~ 0.85, *p* = 0.008) reduction in prediabetes prevalence, respectively, compared to those in Q1 (0.16 ~ 0.98), with a significant *p* value for the trend through quartiles (0.006).

**Table 4:** Association of dietary Se intake with the prevalence of prediabetes.

Dietary Se intake	Model 1		Model 2		Model 3	
	OR (95% CI)	*p* value	OR (95% CI)	*p* value	OR (95% CI)	*p* value
Se intake (μg/kg/d)^a^	0.18 (0.11, 0.29)	<0.001	0.28 (0.16, 0.47)	<0.001	0.15 (0.07, 0.36)	<0.001
Categories						
Q1 (0.16 ~ 0.98)	Reference		Reference		Reference	
Q2 (0.99 ~ 1.34)	0.73 (0.55, 0.97)	0.03	0.81 (0.60, 1.09)	0.16	0.79 (0.57, 1.10)	0.16
Q3 (1.35 ~ 1.82)	0.46 (0.34, 0.63)	<0.001	0.54 (0.39, 0.75)	<0.001	0.52 (0.36, 0.77)	0.001
Q4 (1.83 ~ 6.69)	0.42 (0.31, 0.58)	<0.001	0.57 (0.41, 0.80)	0.001	0.54 (0.35, 0.85)	0.008
*p* for trend	<0.001		<0.001		0.006	
Se intake (μg/d)^a^	0.42 (0.27, 0.71)	0.001	0.55 (0.31, 0.96)	0.04	0.67 (0.25, 1.77)	0.41
Categories						
Q1 (10.60 ~ 72.36)	Reference		Reference		Reference	
Q2 (72.37 ~ 97.54)	0.80 (0.59, 1.07)	0.13	0.83 (0.61, 1.13)	0.23	0.90 (0.64, 1.27)	0.55
Q3 (97.55 ~ 128.28)	0.58 (0.42, 0.80)	0.001	0.63 (0.45, 0.88)	0.01	0.67 (0.45, 1.00)	0.05
Q4 (128.29 ~ 399.63)	0.68 (0.50, 0.93)	0.014	0.79 (0.57, 1.08)	0.14	0.91 (0.57, 1.46)	0.71
*p* for trend	0.007		0.09		0.64	

^a^Dietary Se intake was log-transformed in the multivariate logistic regression analysis. In sensitivity analysis, dietary selenium intake was converted from a continuous variable to a categorical variable (quartiles). Model 1 was crude model that no covariates were adjusted; Model 2 was adjusted for age, and sex; Model 3 was further adjusted for physical activity, caloric intake, family history of diabetes, and history of smoking. Se, selenium; OR, odds ratio; CI, confidence interval.

However, when dietary Se intake was expressed in μg/d, no significant correlations were found between dietary Se intake and the prevalence of prediabetes in the fully adjusted model, as shown in Table 4.

### Dose–response and threshold effect of dietary se intake on prediabetes prevalence

**Figure 2:**
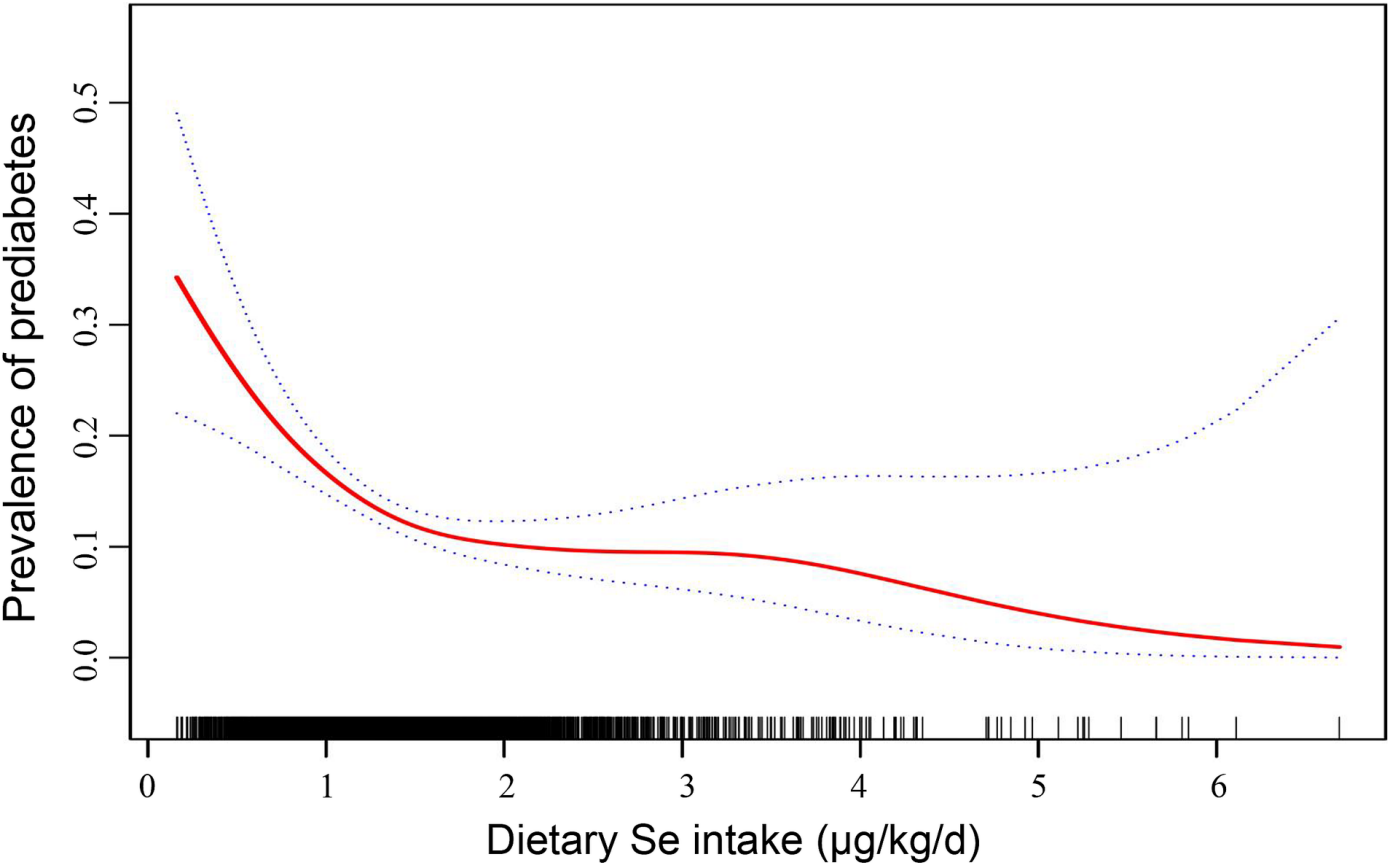
Dose–response relationship between dietary Se intake (μg/kg/d, actual dietary Se intake) and the prevalence of prediabetes. The solid line indicates the estimated prevalence of prediabetes, and the dotted lines represent a 95% CI from the fit. Adjusted for age, sex, physical activity, caloric intake, family history of diabetes, and history of smoking. Se, selenium; CI, confidence interval.

Furthermore, we employed generalized additive modeling and smooth curve fitting to investigate the association between dietary Se intake (μg/kg/d, actual dietary Se intake) and the prevalence of prediabetes. Figure 2 demonstrated a negative non-linear association between dietary Se intake and prediabetes prevalence (*p*<0.001). The threshold effect analysis from two-piecewise linear regression revealed that the inflection point was at 1.42 μg/kg/d (Table 5). Below the inflection point, an significant inverse association was detected between dietary Se intake and prediabetes prevalence (OR = 0.31, 95% CI: 0.19 ~ 0.52, *p*<0.001). However, this association did not reach statistical significance above the inflection point (*p* = 0.25).

**Table 5:** Threshold effect analysis of dietary Se intake (μg/kg/d, actual dietary Se intake) with prediabetes prevalence addressing by two-piecewise regression model.

Models	OR (95% CI)	*p* value
Linear regression model	0.63 (0.49, 0.83)	<0.001
Two-piecewise linear regression model		
Inflection point (1.42 μg/kg/d)		
<1.42 μg/kg/d	0.31 (0.19, 0.52)	<0.001
≥1.42 μg/kg/d	0.84 (0.63, 1.13)	0.25
*p* for logarithmic likelihood ratio test	0.002	

Adjusted for age, sex, physical activity, caloric intake, family history of diabetes, and history of smoking. Se, selenium; CI, confidence interval.

### Subgroup analyses of the association between se intake and prediabetes

**Figure 3:**
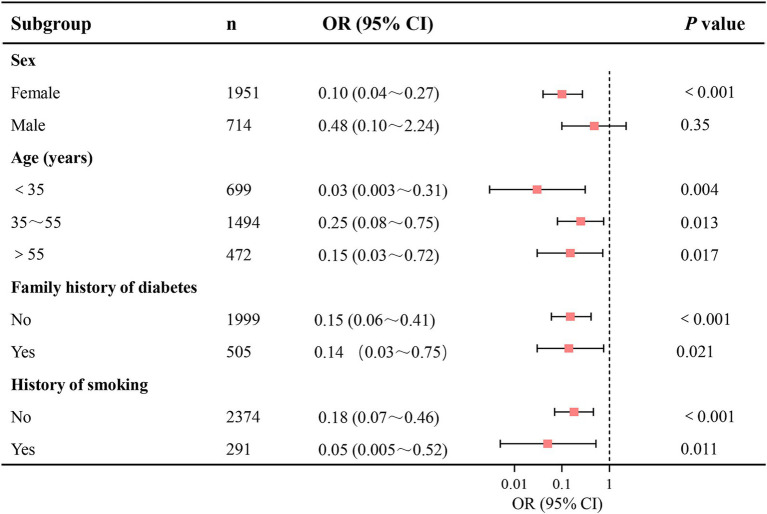
Subgroup analyses of the association between dietary Se intake and the prevalence of prediabetes. Dietary Se intake was log-transformed in the logistic regression analyses. Se, selenium; CI, confidence interval.

Subgroup logistic regression analyses were performed based on the stratified factors, including sex, age (<35, 35 ~ 55, >55), family history of diabetes, and history of smoking. After adjusting for all potential confounders, inverse associations between log-transformed dietary Se intake and prediabetes prevalence were observed across subgroups of age, family history of diabetes, and history of smoking (*p*<0.05 for all; Figure 3). However, the association was statistically significant in females (OR = 0.10, *p*<0.001) but not in males (OR = 0.48, *p* = 0.35). For other subgroups, ORs were as follows: Age <35 years (OR = 0.03, *p* = 0.004), Age 35 ~ 55 years (OR = 0.25, *p* = 0.013), Age >55 years (OR = 0.15, *p* = 0.017), Family history of diabetes (OR = 0.14, *p*<0.001), No family history of diabetes (OR = 0.15, *p*<0.001), History of smoking (OR = 0.05, *p* = 0.011), and No history of smoking (OR = 0.18, *p*<0.001).

## Discussion

Se primarily exerts its biological effects through selenoproteins, which play a crucial role in reducing oxidative stress and inflammation—processes known to contribute to metabolic disorders such as obesity, metabolic syndrome, insulin resistance, and *β*-cell dysfunction (5, 6, 26). Indeed, our previous studies revealed a significant inverse dose-dependent relationship between dietary Se intake (μg/kg/d) and obesity severity, as indexed by various body composition measurements, including BMI, WC, WHR, total body fat%, trunk fat%, android fat%, and gynoid fat% (14). We also observed a negative correlation between Se intake (μg/kg/d) and insulin resistance, as indexed by HOMA-IR (15). Consistent with our prior findings, this study revealed that higher dietary Se intake (μg/kg/d) was associated with improved metabolic profiles. Specifically, increases in Se intake quartiles were paralleled by enhancements in various metabolic markers, including adiposity indicators (BMI, WC, WHR), blood pressure, FPG, lipid profiles, and insulin resistance as assessed by the TyG index. In contrast, absolute Se intake (μg/d) showed inconsistent associations with metabolic parameters in this study. For instance, higher absolute Se intake quartiles exhibited slightly increased WHR and reduced HDL-C, accompanied by elevated body weight, suggesting that unadjusted Se metrics may obscure true biological effects, especially in overweight or obese populations. This can be supported by evidence revealing that Se distribution and selenoprotein activity were disrupted in overweight and obese individuals, particularly reducing glutathione peroxidase antioxidant activity, which impairs Se’s metabolic benefits (27, 28). Thus, weight-adjusted Se intake may better reflect Se bioavailability per metabolic mass, particularly in subjects with overweight and obesity.

Besides, this study demonstrated a graded reduction in prediabetes prevalence across weight-adjusted Se quartiles (19.85% in Q1 vs. 9.61% in Q4). Additionally, we observed an inverse association between dietary Se intake (μg/kg/d) and the prevalence of prediabetes in this Newfoundland cohort. Specifically, for every unit increase in log-transformed dietary Se intake (μg/kg/d), the prevalence of prediabetes decreased by 85% after fully adjusting for age, sex, physical activity, caloric intake, family history of diabetes, and history of smoking. These findings indicate that Se may play a protective role in the development of prediabetes. In line with our results, previous studies have shown that Se supplementation can reduce insulin resistance, decrease serum high-sensitivity C-reactive protein (hs-CRP), and lower biomarkers of oxidative stress in patients with gestational diabetes or polycystic ovary syndrome (29–31). Moreover, a meta-analysis of 20 randomized controlled trials indicated that Se supplementation could reduce fasting insulin levels and improve insulin sensitivity [10], which also supports the beneficial effect of Se on glucose metabolism. However, no significant correlations were found between absolute Se intake (μg/d) and prediabetes in fully adjusted models. This suggests that body weight may influence Se’s metabolic impact, as higher body weight could diminish the apparent benefits of a given absolute Se intake. Thus, weight-adjusted Se intake (μg/kg/d) better captures the relationship with prediabetes by accounting for body size variations. The use of body weight-adjusted Se intake remains methodologically justified for precision in the following dose–response relationships.

Our study further explored the dose–response relationship and threshold effect of actual dietary Se intake on prediabetes prevalence. We found a significant non-linear inverse association between dietary Se intake and prediabetes prevalence below a threshold of 1.42 μg/kg/d. Specifically, for each 1-unit increase in dietary Se intake (μg/kg/d), the prevalence of prediabetes decreased by 69%. However, the association reached no statistical significance beyond this threshold. Our identified threshold (1.42 μg/kg/d) aligns closely with prior studies suggesting a “ceiling effect” for Se’s metabolic benefits. For instance, our previous study reported a negative correlation between dietary Se intake (μg/kg/d) and HOMA-IR in subjects whose dietary Se intake was below 1.6 μg/kg/d, while the negative correlation was no longer significant when dietary Se intake was above 1.6 μg/kg/d (15). Similarly, a cross-sectional study conducted in 128 non-diabetic overweight and obese Malaysian adults showed an inverse association between dietary Se and HOMA-IR with low intake of Se (<1.0 μg/kg/d), whereas a positive association was found in individuals with relatively high intake of Se (≥1.0 μg/kg/d) (32). Additionally, a study using data from CHNS with a cohort of 5,970 participants in China reported a V-shaped relationship between dietary Se intake and diabetes risk. The risk of diabetes decreased as Se intake increased below 45 μg/d, while the risk gradually increased when dietary Se intake surpassed 45 μg/d (9). However, other studies have reported conflicting results. A systematic review and dose–response meta-analysis of 34 non-experimental studies revealed a positive association between dietary Se intake and diabetes risk, indicating that Se intakes of 80 and 120 μg/d were correlated with risk ratios of 1.23 and 1.55 compared with the reference category of 55 μg/d, respectively (33). Consistently, another meta-analysis conducted by the same group earlier also demonstrated a similar increasing trend in the risk of diabetes, which showed that compared with the reference category of 23 μg/d, Se intakes of 50 and 75 μg/d were associated with risk ratios of 1.5 and 1.9, respectively (34). Moreover, a prospective study from Northern Italy (*n* = 7,182) showed that increased dietary Se intake was associated with an increased risk of T2DM, revealing that the OR for diabetes comparing the highest quintile of Se intake (>65.9 μg/d) to the lowest quintile (≤47 μg/d) was 2.39 (7). Similarly, a cross-sectional study included 41,474 adults in the National Health and Nutrition Examination Survey (NHANES 1999–2006) reported that compared to the lowest quartile of dietary Se intake (<59.68 μg/d), the highest quartile (>119.48 μg/d) was associated with an increased risk of diabetes (OR = 2.139) (35). A similar positive correlation was also found in a European cohort with an OR of 1.045 (36). By contrast, cross-sectional studies with Brazilian participants and US adults, as well as a Chinese study including patients with chronic pancreatitis, found no association between dietary Se intake and diabetes/prediabetes (8, 37, 38). These discrepancies may be attributed to differences in study populations with different genetic backgrounds and environmental factors. The Newfoundland population, characterized by genetic isolation and high obesity rates (39.4%), may further differentiate these associations. Additionally, whether the studies used body weight-adjusted Se intake (μg/kg/d) or absolute Se intake (μg/d) may also contribute to the inconsistency.

Subgroup analyses in this study demonstrated consistent inverse associations between log-transformed dietary Se intake and prediabetes across age groups, family history of diabetes, and history of smoking. These findings reinforce the robustness of Se’s protective role, irrespective of these factors. However, the association was statistically significant in females (OR = 0.10, *p* < 0.001) but not in males (OR = 0.48, *p* = 0.35), suggesting a sex-specific divergence. The lack of statistical significance in the male subgroup may stem from several sex-specific biological and behavioral factors. Biologically, females often show enhanced expression and function of selenoproteins due to estrogen-mediated upregulation of glutathione peroxidase, amplifying Se’s antioxidant and glucose-regulatory effects (39–41). In males, however, lower baseline testosterone levels and elevated estradiol (linked to insulin resistance)—common in prediabetes—may impair metabolic responses to Se, and further attenuate Se’s benefits (42, 43). Behaviorally, males tend to have poorer baseline dietary patterns and lower adherence to nutrient-rich foods (e.g., vegetables, whole grains). These patterns, combined with sex-specific confounders such as dyslipidemia (more prevalent in middle-aged men) and central obesity, may obscure Se’s protective effects (44–46). Future research should further investigate sex-disparate mechanisms in Se metabolism, including hormonal interactions and lifestyle confounders.

However, our study has several limitations. The cross-sectional design of this study limits the ability to establish causality. Thus, longitudinal studies are needed to confirm the causal relationship between dietary Se intake and prediabetes development. While FFQs provide cost-effective dietary data, recall bias may attenuate the observed associations. More accurate methods, such as biomarkers of Se status, could improve the validity of the findings. Residual confounding by unmeasured factors, such as socioeconomic status, cannot be excluded despite adjusting for multiple potential confounders in this study.

## Conclusion

The present study demonstrates a significant inverse association between body weight-adjusted dietary Se intake (μg/kg/d) and prediabetes prevalence in a genetically isolated Newfoundland population with high obesity rates. A non-linear threshold effect was identified at 1.42 μg/kg/d, with a 69% reduction in prediabetes risk per unit increase in Se intake below this threshold. Beyond this threshold, no significant additional benefit was observed, suggesting a ceiling effect for Se’s metabolic protection. Crucially, absolute Se intake (μg/d) showed no significant correlation with prediabetes after adjustment, underscoring that body weight adjustment is essential to reveal Se’s true biological impact, particularly in overweight/obese populations where selenoprotein activity may be disrupted. The protective association was consistent across age groups, family history of diabetes, and smoking status but exhibited sex-specific divergence: it was statistically significant in females but not in males. This highlights the need to explore sex-disparate mechanisms in Se metabolism. These findings emphasize that dietary Se recommendations and strategies targeting prediabetes prevention should account for body weight, especially in high-obesity populations. Future longitudinal studies using precise Se biomarkers are warranted to confirm causality and refine optimal intake thresholds.

## Data Availability

The raw data supporting the conclusions of this article will be made available by the authors, without undue reservation.

## Footnotes

^1^ http://www.empowerstats.com

## Glossary

T2DM: Type 2 Diabetes Mellitus
ADA: American Diabetes Association
IFG: Impaired Fasting Glucose
IGT: Impaired Glucose Tolerance
HbA1C: Glycated Hemoglobin
BMI: Body Mass Index
WC: Waist Circumference
HC: Hip Circumference
WHR: Waist-to-Hip Ratio
SBP: Systolic Blood Pressure
DBP: Diastolic Blood Pressure
FPG: Fasting Plasma Glucose
TG: Triacylglycerols
TC: Total Cholesterol
HDL-C: High-Density Lipoprotein Cholesterol
LDL-C: Low-Density Lipoprotein Cholesterol
TyG: Triglyceride-Glucose Index
HOMA-IR: Homeostasis Model Assessment of Insulin Resistance
DXA: Dual-Energy X-ray Absorptiometry
FFQ: Food Frequency Questionnaire
Se: Selenium
