# Supplementary material for: Association between dietary selenium intake and the prevalence of prediabetes in Newfoundland population: a cross-sectional study
Source: Front Nutr. 2025 Aug 26;12:1615462. doi: 10.3389/fnut.2025.1615462 (PMC12417185; doi:10.3389/fnut.2025.1615462)
Supplement: Supplementary file 1 [file Table_1.doc]

Supplementary Material

# Supplementary Tables

# Supplementary Table 1 Comparisons of key characteristics between the final included participants and excluded participants with missing covariates.

| **Variables** | **Final** **included participants** | **Excluded participants with missing covariates** | ***P*** |
| --- | --- | --- | --- |
|  | **(n=2665)** | **(n=267)** | **value** |
| **Age (years)** | 43.28 ± 0.24 | 44.29 ± 0.88 | 0.27 |
| **Sex, n (%)** |  |  | 0.35 |
| **Male** | 714 (26.79%) | 79 (29.59%) |  |
| **Female** | 1951 (73.21%) | 188 (70.41%) |  |
| **Caloric intake (kcal/d) ^a^** | 1952.49 ± 16.32 | 1993.85 ± 58.33 | 0.45 |
| **FPG (mmol/L)** | 5.02 ± 0.01 | 5.08 ± 0.04 | 0.10 |
| **Dietary Se intake (μg/d) ^a^** | 106.20 ± 1.00 | 101.91 ± 2.92 | 0.17 |

**^a^** Caloric intake and dietary Se intake were log-transformed to normalize distributions for subsequent independent Student’s t-test, however, for descriptive purposes, they were presented as mean ± SE in their original units. FPG, fasting plasma glucose; Se, selenium.
